# Incidence of hypothyroidism after treatment for breast cancer—a Danish matched cohort study

**DOI:** 10.1186/s13058-020-01337-z

**Published:** 2020-10-13

**Authors:** Anne Mette Falstie-Jensen, Buket Ö. Esen, Anders Kjærsgaard, Ebbe L. Lorenzen, Jeanette D. Jensen, Kristin V. Reinertsen, Olaf M. Dekkers, Marianne Ewertz, Deirdre P. Cronin-Fenton

**Affiliations:** 1 Department of Clinical Epidemiology, Aarhus University Hospital, Olof Palmes Allé 43-45, DK-8200 Aarhus N, Denmark; 2 Department of Oncology, Odense University Hospital, Odense, Denmark; 3 Institute of Clinical Research, University of Southern Denmark, Odense, Denmark; 4 National Advisory Unit on Late Effects after Cancer Treatment, Department of Oncology, Oslo University Hospital, Oslo, Norway; 5 Department of Epidemiology, Leiden University Medical Center, Leiden, The Netherlands

**Keywords:** Breast cancer, Hypothyroidism, Matched cohort study, Radiation therapy, Chemotherapy, Late effect, Cancer survivorship

## Abstract

**Background:**

Breast cancer survivors (BCS) may have increased risk of hypothyroidism, but risk according to treatment modality is unclear. We estimated the incidence of hypothyroidism in women with breast cancer, and according to cancer treatment.

**Methods:**

Using nationwide registries, we identified all Danish women aged ≥ 35 years diagnosed with non-metastatic breast cancer (1996–2009). We matched up to five cancer-free women (controls) for each BCS. We excluded women with prevalent thyroid disease. Cancer treatment was chemotherapy with or without radiotherapy (RT) targeting the breast/chest wall only, or also the lymph nodes (RTn). We identified hypothyroidism using diagnostic codes, and/or levothyroxine prescriptions. We calculated the cumulative incidence, incidence rates (IR) per 1000 person-years, and used Cox regression to estimate hazard ratios (HR) and associated 95% confidence intervals (CIs) of hypothyroidism, adjusting for comorbidities.

**Results:**

We included 44,574 BCS and 203,306 matched controls with 2,631,488 person-years of follow-up. BCS had a slightly higher incidence of hypothyroidism than controls [5-year cumulative incidence, 1.8% (95%CI = 1.7–1.9) and 1.6% (95%CI = 1.5–1.6), respectively]. The overall IR was 4.45 (95%CI = 4.25–4.67) and 3.81 (95%CI = 3.73–3.90), corresponding to an adjusted HR = 1.17 (95%CI = 1.11–1.24). BCS who received RTn with chemotherapy (HR = 1.74, 95%CI = 1.50–2.02) or without chemotherapy (HR = 1.31, 95%CI = 1.14–1.51) had an elevated risk of hypothyroidism compared with matched controls and compared with BCS who underwent surgery alone [HR = 1.71, 95%CI = 1.45–2.01 and HR = 1.36, 95%CI = 1.17–1.58, respectively].

**Conclusions:**

BCS have an excess risk of hypothyroidism compared with age-matched controls. BCS and those working in cancer survivorship settings ought to be aware that this risk is highest in women treated with radiation therapy to the lymph nodes and chemotherapy.

## Background

Over the past 30 years, breast cancer survival has improved substantially due to earlier detection and improved adjuvant therapies [1]. Today, almost 90% of patients with early-stage breast cancer receive (neo) adjuvant treatment; 10-year life expectancy after diagnosis is about 75% [2]. Therefore, it is critical to identify harmful late effects of cancer treatment, such as hypothyroidism.

Hypothyroidism affects approximately 3% of the European population. The prevalence of hypothyroidism is higher in women and risk increases with age [3]. If left untreated, hypothyroidism has a substantial negative impact on patient wellbeing and is associated with symptoms like tiredness, weakness, weight gain, and mental changes [4]. Hypothyroidism is characterized biochemically by elevated thyroid-stimulating hormone and normal or low free T_4_ levels in the serum. Hypothyroidism can be controlled with levothyroxine substitution therapy.

Hypothyroidism is a well-documented late effect of radiation therapy (RT) to the lower neck in lymphoma and head and neck cancer patients [5, 6]. Research suggests a higher risk of hypothyroidism among patients with breast cancer, not only among breast cancer patients who underwent RT, but also those treated with systemic therapies [7–19]. Yet, much of the published studies were limited by small sample size and, accordingly, few cases of hypothyroidism. Two large studies (including over 16,000 women) suggested a higher risk of hypothyroidism in breast cancer survivors compared with women without breast cancer [7, 14]. Yet, one was restricted to older women (aged 65+), who may have inherently increased risk of hypothyroidism, and observed no modification in the risk of hypothyroidism according to the extent of RT [14]. The other study had limited information on breast cancer characteristics and failed to evaluate risk according to the extent of RT (i.e. RT to the breast/chest wall only or also incorporating the supraclavicular lymph nodes) [7]. This is an important aspect to explore as several of the smaller studies suggest an elevated risk of hypothyroidism in patients treated with RT to the supraclavicular lymph nodes compared with RT to the breast/chest wall only and that this risk increased with increasing radiation dose [13, 16, 18]. Thus, the impact of cancer-directed treatment modalities on the subsequent risk of hypothyroidism in breast cancer survivors requires clarification.

We therefore evaluated the incidence of hypothyroidism in breast cancer survivors and determined if this risk is modified by treatment modalities, compared with age-matched cancer-free women from the general population.

## Methods

This study was approved by the Danish Medicines Agency and the Danish Breast Cancer Group (DBCG). The use of personal data for the project has been approved by Aarhus University in accordance with General Data Privacy Regulations and the Danish data protection legislation and does not require informed consent from the research subjects or approval from governmental or ethical bodies.

### Design and study population

This population-based matched cohort study used prospectively collected data from national medical registries covering the entire Danish population of 5.7 million inhabitants. All citizens have free access to health care because it is completely tax funded. At birth or immigration, citizens are assigned a unique civil personal registration number that enables accurate and unambiguous individual-level linkage across all public registries [20].

From the DBCG, we assembled a cohort of all women diagnosed with a first-time hospital diagnosis of non-metastatic breast cancer aged ≥ 35 years between January 1, 1996, and December 31, 2009. The DBCG clinical database was established in 1977 to optimize breast cancer diagnosis and treatment across Denmark and to improve breast cancer prognosis [21]. All patients with invasive breast cancer are registered in the DBCG and registration completeness is high (~ 95%) [22]. Treating physicians are responsible for entering pre-specified data on patient, tumour, and treatment characteristics. Breast cancer survivors on treatment protocols have routine follow-up clinic visits twice per year for the first 5 years and annually up to 10 years after diagnosis. In this study, we included all patients diagnosed with breast cancer during the study period and not only those on a treatment protocol [23].

By linking to various registries, we excluded women with prevalent hypothyroidism, hyperthyroidism, and/or previous cancers at breast cancer diagnosis (see codes below).

We used the DBCG database for information on adjuvant treatment, including chemotherapy (yes or no) and RT [(yes or no), and stratified into three categories no, chest wall/breast only (RTc), or with addition of ipsilateral supraclavicular and axillary lymph nodes (RTn)].

### Matched cohort

Using the Danish Civil Registration System, for each breast cancer survivor, we matched with replacement [24] up to five cancer-free women from the general population (hereafter matched controls) with the same year of birth and municipality of residence on the date of breast cancer diagnosis (index date), the latter to account for differences in iodine intake in Denmark [25]. As for the breast cancer cohort, we excluded controls with prevalent hypothyroidism and hyperthyroidism.

### Outcome

Hypothyroidism was defined as a diagnostic code of hypothyroidism (International Classification of Diseases (ICD) 8th edition codes: 244.00-244.03, 244.08, and 244.09, and ICD-10 codes: E03.2-E03.9 and E89.0) and/or at least two redeemed levothyroxine prescriptions in the absence of concomitant anti-thyroid drugs (Anatomical Therapeutic Chemical (ATC) Classification code: H03A) during follow-up. Information on diagnostic codes was obtained from the Danish National Registry of Patients (DNRP) covering information on all discharge diagnoses for inpatient hospital admissions since 1977 and outpatient and emergency room hospital contacts since 1995 [26]. We ascertained information on prescriptions from the Danish National Prescription Registry (DNPreR) covering all dispensed prescriptions since 1995 [27]. The date for hypothyroidism was determined from the date of either a diagnostic code or a redeemed prescription, whichever came first.

### Covariates

We ascertained information on hyperthyroidism from the DNRP (ICD-8 codes: 242.01-242.29 and ICD-10: E05-E05.9 and E05.0B, and/or at least two prescriptions of anti-thyroxine medication during follow-up by ATC-codes: H03BB01, H03BB02, and H03BA02 captured in the DNPreR).

Information on prior cancers was ascertained from the DNRP (ICD-8 codes: 140-209 and ICD-10 codes: C00-99).

We collected information on comorbidities diagnosed up to 10 years before the index date from the DNRP. We evaluated comorbidity for each individual using a modified version of the Charlson comorbidity index (CCI) excluding cancer from the index score [28] and categorized as no comorbidities (CCI = 0), low (CCI = 1 or 2), and high (CCI ≥ 3).

For the breast cancer cohort, we retrieved information on clinical characteristics from the DBCG including menopausal status at diagnosis, tumour size, histological grade, lymph node status, tumour oestrogen receptor (ER) status, and human epidermal growth factor receptor 2 (HER-2) status. We derived cancer stage by combining information on tumour size and lymph node status.

### Statistical analyses

We present descriptive statistics as count and percentages for the breast cancer cohort and matched control cohort, and tumour and treatment characteristics for the breast cancer cohort. We summarized the data sources for identifying cases of hypothyroidism (i.e. prescription data versus hospital diagnoses—incorporating the main admission diagnosis and any supplementary diagnoses) in each cohort. We calculated the cumulative incidence, incidence rates (IRs) of hypothyroidism per 1000 person-years, and associated 95% confidence intervals (95%CIs), and stratified results by calendar period, age, and CCI. To explore the risk of hypothyroidism in strata of cancer treatment, we created a composite variable with six categories for the receipt of RT and chemotherapy (CT) (RT−/CT−, RT−/CT+, RTc/CT−, RTc/CT+, RTn/CT−, or RTn/CT+). IRs of hypothyroidism with 95%CIs were computed for each category in the breast cancer cohort and the matched control cohort. We plotted the cumulative incidence of hypothyroidism in the breast cancer and matched control cohorts and stratified according to treatment modality.

Using Cox proportional hazards regression models using time since diagnosis/index date as the underlying time scale, we estimated hazard ratios (HRs) with 95%CI comparing the breast cancer cohort with the matched cohort adjusting for age and the CCI at the diagnosis/index date. This approach was repeated in strata of treatment modalities. Within the breast cancer cohort, we estimated HRs using no treatment (RT−/CT−) as the reference group. The models accounted for competing risks of death, emigration, and hyperthyroidism. We checked the proportional hazard assumption by visual inspection of the log of the estimated survivor function in the model. Follow-up time began 6 months after breast cancer diagnosis/index date to ensure completion of primary treatment. Follow-up time continued from 6 months after diagnosis/index through 1 January 2016.

We conducted several sensitivity analyses. We stratified our analyses by the CCI to clarify if individuals with high comorbidity had a higher IR of hypothyroidism than those with low comorbidity due to more regular contact with healthcare providers. We also stratified by age and calendar to assess if the association changed by age or over calendar time. In the analyses restricted to the breast cancer cohort, we additionally adjusted for cancer stage, grade, and ER status (in addition to age and comorbidity).

All statistical analyses were performed using SAS version 9.4 (SAS Institute, Cary, NC).

## Results

The study cohorts consisted of 44,574 breast cancer survivors and 203,306 matched controls with 384,401 and 2,082,014 person-years of follow-up, respectively (Supplementary Figure 1). Median follow-up was 8.4 years (interquartile range 5.6, 12.6) and 10.3 years (interquartile range 7.1, 14.2) in each cohort, respectively. The mean age at diagnosis/index for both cohorts was 61 years. Breast cancer survivors had a higher comorbidity burden than matched controls (Table 1). In both cohorts, 4% of hypothyroidism was identified by a hospital diagnosis code only, 64% by a redeemed prescription only, and 32% by a hospital diagnosis code and redeemed prescription.

**Table 1:** Baseline characteristics of women diagnosed with non-metastatic breast cancer between 1996 and 2009 who were registered in the Danish Breast Cancer Group clinical database, and a matched control cohort of cancer-free women from the general population

	Breast cancer survivors		Matched controls	
	Numbers	%	Numbers	%
**Total**	44,574	100.0	203,306	**100.0**
**Calendar year of diagnosis**				
1996–1999	11,261	25.3	52,931	26.04
2000–2003	12,285	27.6	56,836	27.96
2004–2006	9458	21.2	42,819	21.06
2007–2009	11,570	26.0	50,720	24.95
**Age at breast cancer diagnosis, years**				
35–39	1367	3.1	6550	3.22
40–49	7123	16.0	34,454	16.95
50–59	12,543	28.1	58,850	28.95
60–69	12,945	29.0	58,230	28.64
70–79	7704	17.3	33,091	16.28
80 or older	2892	6.5	12,131	5.97
**Charlson comorbidity index (modified)**^**1**^				
No	35,832	80.39	173,470	85.32
Low	7083	15.89	27,111	13.34
High	1659	3.72	2725	1.34
**Menopausal status at diagnosis**				
Unknown	62	0.1		
Premenopausal	10,833	24.3		
Postmenopausal	33,679	75.6		
**Tumour size, mm**				
Unknown	2263	5.08		
Under 21	24,622	55.24		
21–50	16,033	35.97		
Over 50	1656	3.72		
**Lymph node status, numbers**				
Unknown	2903	6.51		
N0	21,757	48.81		
N1–3	12,531	28.11		
N4+	7383	16.56		
**UICC stage**				
Unknown	3244	7.3		
I	15,357	34.5		
II	18,246	40.9		
III	7727	17.3		
**Histological grade**				
Unknown	8376	18.79		
Low	11,664	26.17		
Moderate	16,206	36.36		
High	8328	18.68		
**ER status**				
Unknown	2562	5.75		
ER negative (0–9%)	8320	18.67		
ER positive (≥ 10%)	33,692	75.59		
**HER2 status**^**2**^				
Unknown	28,005	62.83		
Negative	13,429	30.13		
Positive	3140	7.04		

^**1**^Charlson comorbidity index (CCI) without cancer included (low: score of 1 or 2; high: score of 3 or more)

^2^Systematic recording of HER-2 status started in 2007

### Cumulative incidence, incidence rate, and hazard ratio of hypothyroidism overall

**Fig. 1:**
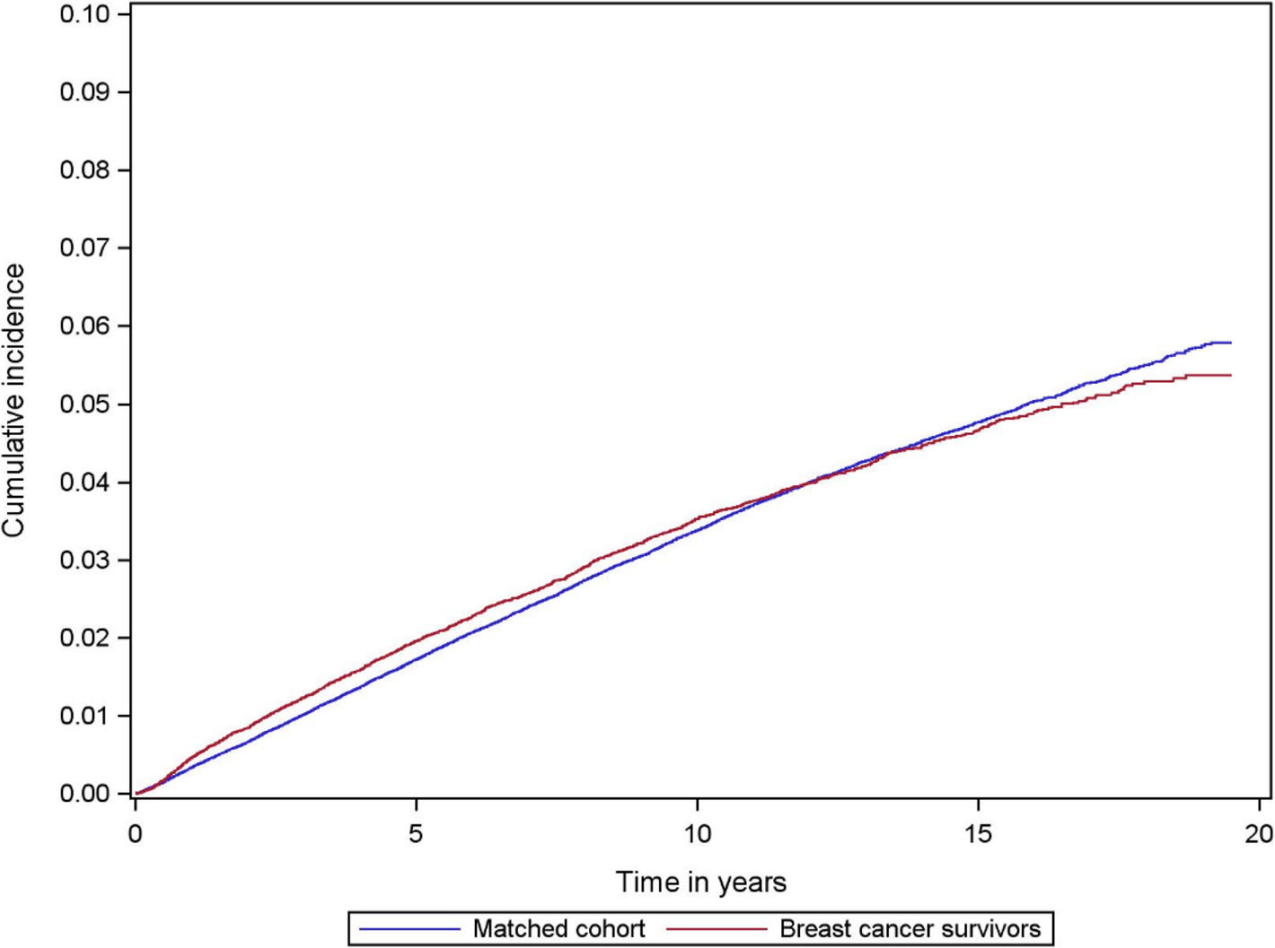
Cumulative incidence curves of hypothyroidism in the cohort of Danish women diagnosed with non-metastatic breast cancer 1996–2009 and a matched control cohort of cancer-free women

During follow-up, 1712 breast cancer survivors and 7936 matched controls developed hypothyroidism. Five years after breast cancer diagnosis or index date (control cohort), the cumulative incidence of hypothyroidism was 1.8% (95%CI = 1.7 to 1.9) and 1.6% (95%CI = 1.5 to 1.6) in the breast cancer and control cohorts, respectively. The cumulative incidence of hypothyroidism was higher up to approximately 12 years after diagnosis, from which point the incidence was higher in the matched control cohort (Fig. 1). The cumulative person-time was similar in both cohorts up to approximately 9 years after diagnosis/index, at which point it was higher in the matched control cohort (Supplementary Figure 2).

**Fig. 2:**
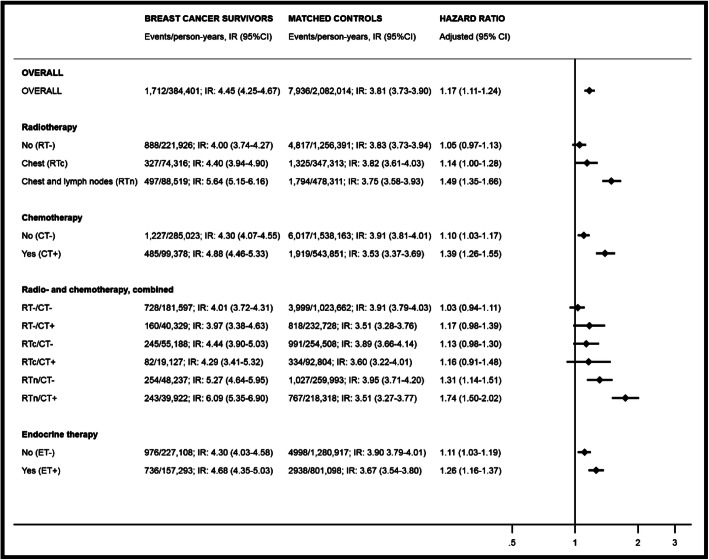
Incidence rates (IRs), hazard ratios, and associated 95% confidence intervals (95%CIs) of hypothyroidism in the cohort of Danish women diagnosed with non-metastatic breast cancer 1996–2009 and matched control cohort of cancer-free women. IRs and HRs (HRs adjusted for Charlson comorbidity index) overall, and stratified by treatment modalities. RT, radiation therapy to the chest wall/breast (RTc), or with addition of the lymph nodes (RTn); CT, chemotherapy

The overall IR for hypothyroidism was higher in the breast cancer cohort than in the matched control cohort (IR = 4.45; 95%CI = 4.25 to 4.67 versus 3.81; 95%CI = 3.73 to 3.90, respectively) (Fig. 2). Compared with the matched controls, breast cancer survivors were more likely to develop hypothyroidism during follow-up (adjusted HR = 1.17; 95%CI = 1.11 to 1.24). This result remained robust to stratification by calendar period, age, and comorbidity status (Supplementary Table 1).

### Incidence rate and hazard ratio of hypothyroidism according to treatment modality

**Fig. 3:**
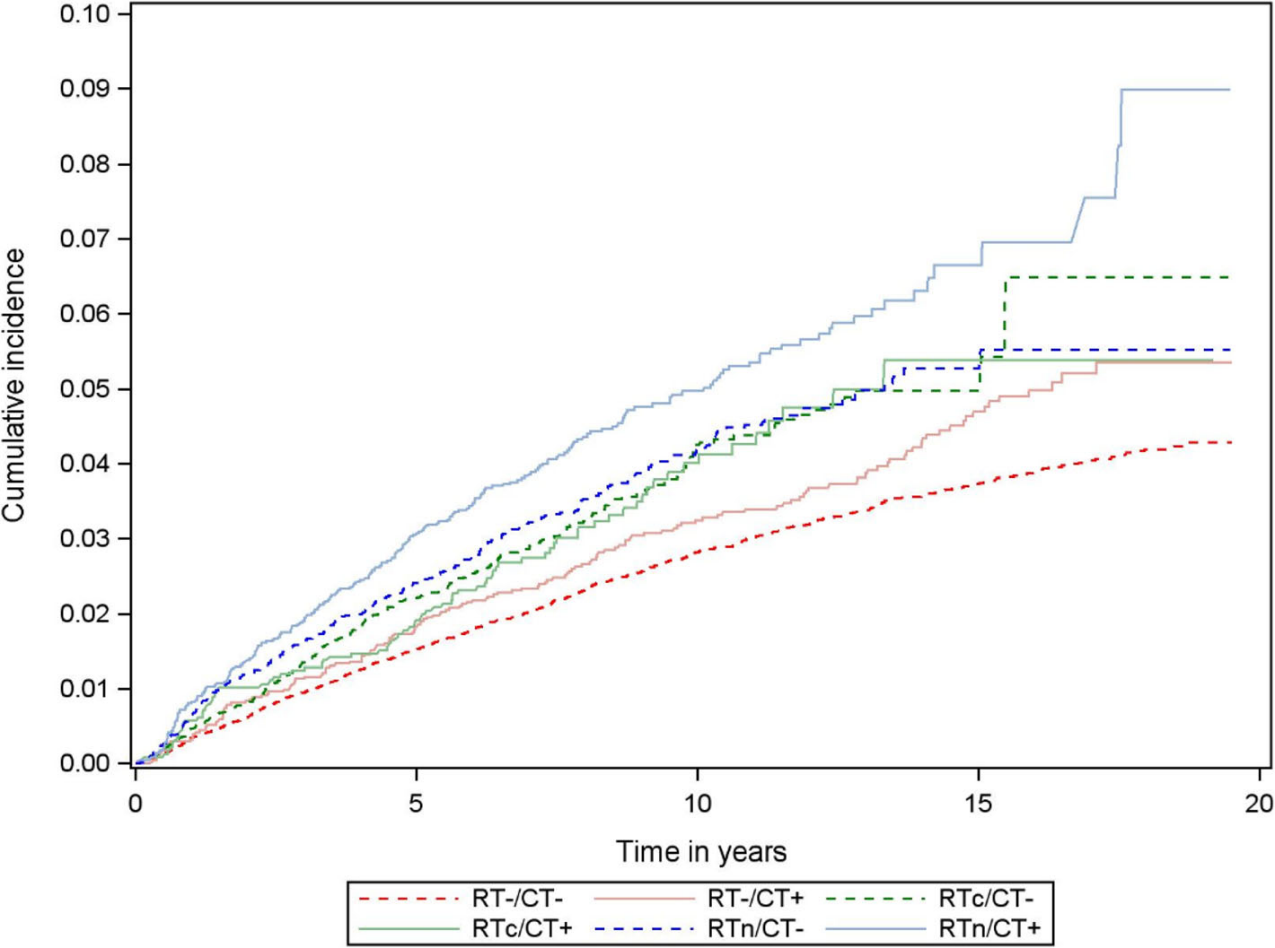
Cumulative incidence curves of hypothyroidism in a cohort of Danish women diagnosed with non-metastatic breast cancer 1996–2009, according to the receipt of radiation therapy—to the breast/chest wall only (RTc) or with the addition of the lymph nodes (RTn)—and chemotherapy (CT)

Although breast cancer survivors had an increased risk of hypothyroidism, the risk of hypothyroidism differed according to treatment modality. Breast cancer survivors who received RTn or chemotherapy had an increased risk of hypothyroidism compared with matched controls (RTn: adjusted HR = 1.49; 95%CI = 1.35 to 1.66, and chemotherapy: adjusted HR = 1.39; 95%CI = 1.26 to 1.55). The HRs were similar to the overall breast cancer cohort among women who did and did not receive endocrine therapy (Fig. 2). We observed the highest rates of hypothyroidism in breast cancer survivors who received both RTn and chemotherapy (Figs. 2 and 3): adjusted HR = 1.74 (95%CI = 1.50 to 2.02).

### Hazard ratio of hypothyroidism: a within breast cancer cohort comparison

In analyses restricted to the breast cancer cohort alone, RTn with or without chemotherapy was associated with an elevated risk of hypothyroidism compared with not receiving these therapies (Table 2). Women who received RTn had the highest risk of hypothyroidism (adjusted HR without chemotherapy, 1.36; 95%CI = 1.17 to 1.58, and adjusted HR with chemotherapy, 1.71; 95%CI = 1.45 to 2.01) compared with those who did not undergo RT or chemotherapy. Additional adjustment for cancer stage, grade, and ER status did not materially change the magnitude or direction of the HRs (Supplementary Table 2).

**Table 2:** Incidence rates (IRs) and hazard ratios and associated 95% confidence intervals (95%CIs) of hypothyroidism in the cohort of women diagnosed with non-metastatic breast cancer between 1996 and 2009, who were registered in the Danish Breast Cancer Group clinical database, stratified by the receipt of radiation therapy—to the chest wall only (RTc) or with the addition of the lymph nodes (RTn)—and chemotherapy

		Breast cancer survivors				Hazard ratio	
Treatment modalities	Median age (years)	Cases of hypothyroidism	Numbers	Person years	IR pr. 1000 PY (95% CI)	Crude (95% CI)	Adjusted^1^ (95% CI)
RT−/CT−	66.8	728	20,754	181,597	4.01 (3.72–4.31)	Reference group	
RT−/CT+	50.1	160	4042	40,329	3.97 (3.38–4.63)	0.99 (0.83–1.17)	1.10 (0.91–1.32)
RTc/CT−	62.1	245	6615	55,188	4.44 (3.90–5.03)	1.12 (0.96–1.29)	1.15 (0.99–1.34)
RTc/CT+	51.8	82	2252	19,127	4.29 (3.41–5.32)	1.08 (0.86–1.36)	1.19 (0.94–1.51)
RTn/CT−	62.5	254	6065	48,237	5.27 (4.64–5.95)	1.32 (1.14–1.53)	1.36 (1.17–1.58)
RTn/CT+	50.1	243	4846	39,922	6.09 (5.35–6.90)	1.53 (1.32–1.77)	1.71 (1.45–2.01)

*PY* person years, *CI* confidence interval, *RT* radiotherapy, *CT* chemotherapy, *RTc* radiotherapy to the chest wall only, *RTn* radiotherapy to the chest wall with addition of lymph nodes (supraclavicular, axillary)

^1^Adjusted for age and comorbidity at diagnosis

## Discussion

In this matched cohort study, we observed a higher risk of hypothyroidism in breast cancer survivors compared with cancer-free women. This excess risk of hypothyroidism was evident irrespective of treatment modality but was particularly high in breast cancer patients who received RT targeting the lymph nodes, with the highest risk among those who also received chemotherapy. Our data suggests that this elevated risk of hypothyroidism in breast cancer survivors was evident as early as the first year after diagnosis. At approximately 12 years after breast cancer diagnosis/index date, the cumulative incidence of hypothyroidism was higher in the matched control cohort, likely reflecting the excess mortality in the breast cancer cohort compared with the matched control cohort.

Our findings are consistent with those in a Norwegian study, which reported over twofold excess risk of hypothyroidism in a breast cancer cohort [13]. That study was restricted to women with stage II and III breast cancer (median age 51 years) who had received RT targeting the lymph nodes, most of whom also received chemotherapy. Information on hypothyroidism was obtained via self-report so the study may be prone to outcome misclassification. Although our overall excess risk of hypothyroidism was lower than that observed in the Norwegian study, our study population included patients with stage I disease, who are generally not recommended RT to the lymph nodes. Furthermore, we observed the highest risk of hypothyroidism among patients who received RT to the lymph nodes and chemotherapy: a subgroup of patients comparable in age and cancer treatment to those in the Norwegian study.

Registry-based studies from Canada, the US, and the UK demonstrated an increased risk of hypothyroidism among breast cancer survivors, with relative effect estimates similar to those observed in our study (HRs of 1.19, 1.21, and 1.26, respectively) [7, 14, 19]. However, the US and UK studies ascertained information on hypothyroidism using diagnostic codes only without incorporating prescription data. We note that in our Danish setting, information on hypothyroidism was obtained predominantly via prescriptions for levothyroxine rather than diagnostic codes from hospital admissions. The US study included beneficiaries of Medicare, which is primarily available to individuals aged 65 years and above. Although the age distribution of participants in the UK study was similar to that in our study, they had a substantially higher prevalence of comorbid conditions, which could influence the likelihood of a diagnosis of hypothyroidism [7]. The UK study reported the incidence of hypothyroidism among 5-year survivors of breast cancer, which may restrict comparison with our findings. In our study, breast cancer survivors with comorbid diseases had a higher risk of hypothyroidism compared with those without comorbidities. Though the increased risk of hypothyroidism was evident within the same comorbidity strata in the matched control cohort, we cannot eliminate the possibility that surveillance bias influenced our findings due to more regular contact with healthcare services in the cancer survivor cohort compared with the general population. A limitation of the aforementioned registry-based studies is that none incorporated information on cancer-directed treatment. They were therefore unable to pinpoint a patient group with the highest risk of hypothyroidism.

Breast cancer RT may cause incidental exposure to adjacent tissues and has been associated with an increased risk of ischemic heart disease [29]. Given the proximity of the thyroid gland to the supraclavicular lymph nodes, we hypothesized that RT to these lymph nodes would confer the highest risk of incidental thyroid irradiation causing thyroid injury—manifesting as hypothyroidism. Though we had no information on the actual RT dose to the thyroid gland, the increased risk of hypothyroidism with more extensive RT supports our hypothesis. Preclinical studies suggest that chemotherapy may alter levels of thyroid hormone-binding proteins, but without clinical impact [30]. Our findings of no excess risk of hypothyroidism among women treated with chemotherapy without RT compared with those who did not receive chemotherapy seem consistent with this research. Nonetheless, chemotherapy may sensitize the thyroid gland to RT [6]. This may explain our finding of the highest risk of hypothyroidism among patients who underwent chemotherapy in addition to RT to the lymph nodes.

Younger breast cancer patients tend to have poorer prognosis and generally receive more aggressive cancer-directed treatment than older patients [31–33]. Accordingly, we note that the median age was lower among women who received chemotherapy compared with those who did not, irrespective of the receipt of RT. We also observed the highest relative risk of hypothyroidism in the youngest patient group compared with their cancer-free counterparts, and the magnitude of the relative risk decreased with increasing age. These findings suggest that the overall excess risk of hypothyroidism in the breast cancer cohort compared with the control cohort is likely attributable to the receipt of cancer-directed treatment rather than breast cancer per se.

Primary strengths of our study are the large sample size and long-term complete follow-up, the use of comprehensive clinical data on breast cancer patients, and the treatment information on all subjects. Individual-level data linkage to the Danish population-based registries enabled inclusion of detailed information on comorbid and other thyroid diseases for all participants. In addition, we were also able to include information on redeemed prescriptions. We excluded patients with prevalent hyperthyroidism at breast cancer diagnosis/index date. We also censored patients who developed hyperthyroidism during follow-up, as some treatments for hyperthyroidism such as radioactive iodine and surgery may increase the risk of hypothyroidism [34].

Among our study’s limitations are the risk of misclassification in relation to outcome status because hypothyroidism is underdiagnosed in the general population [3]. Such disease misclassification may be differential, given the potential for more frequent healthcare contact in cancer patients (i.e. surveillance bias). This may have increased our effect estimates. Importantly, we had no data on serum thyroid hormone levels or the presence of autoimmune antibodies, which may have helped to further classify the underlying cause and severity of the hypothyroidism. Nonetheless, we are unaware of any evidence confirming a common aetiology of autoimmune thyroiditis and breast cancer.

We had no information on lifestyle factors, so our findings may be prone to unmeasured confounding from factors such as smoking, obesity, and physical activity [14, 35]. However, we considered comorbidity to be a proxy for poor health. The similarity of our crude and adjusted estimates points to a low likelihood of unmeasured confounding. We defined hypothyroidism as the redemption of at least two levothyroxine prescriptions. In Denmark, patients pay for a proportion of the cost of redeemed prescriptions so prescription redemption is likely to reflect intention to adhere with the prescription and therefore a robust measure.

It is worth noting that cancer-directed treatments in our study were based on intention-to-treat data from DBCG, rather than received treatments. Furthermore, we did not incorporate information on breast cancer recurrence in this study or on treatments given for recurrent breast cancer. It is likely that patients diagnosed with recurrent breast cancer, or other new primary cancers, would be treated with radiation therapy and/or chemotherapy. This may have contributed to our observed elevated risk of hypothyroidism in the breast cancer compared with the comparison cohort.

## Conclusions

Our large prospective study suggests that women with breast cancer had a long-term elevated risk of hypothyroidism compared with matched cancer-free women from the general population. The risk of hypothyroidism was highest in breast cancer survivors treated with RT targeting the lymph nodes, especially among those who also received chemotherapy. The thyroid gland should be considered an organ at risk in breast cancer radiation therapy targeting the lymph nodes and the dose to the thyroid gland minimized when possible. Oncologists and health care professionals working in cancer survivorship settings, as well as breast cancer survivors themselves, ought to be vigilant to the signs and symptoms of hypothyroidism, particularly among survivors who received radiation therapy to the lymph nodes and chemotherapy.

## Supplementary information

**Additional file 1: Supplementary Figure 1.** CONSORT diagram showing the of inclusion and exclusion criteria for the cohort of breast cancer survivors and the matched control cohort. **Supplementary Table 1.** Incidence rates (IRs) and hazard rates§ of hypothyroidism for survivors of non-metastatic breast cancer and matched controls in strata by calendar period, age and comorbidity at index date. **Supplementary Figure 2.** Cumulative person-time in the cohort of survivors of non-metastatic breast cancer and the matched control cohort, with that in the matched controls divided by 5 to account for the 1:5 matching. **Supplementary Table 2.** Incidence rates (IRs) and hazard ratios and associated 95% confidence intervals (95%CI) of hypothyroidism in the cohort of women diagnosed with non-metastatic breast cancer between 1996 and 2009, who were registered in the Danish Breast Cancer Group clinical database, stratified by the receipt of radiation therapy – to the chest wall only (RTc) or with the addition of the lymph nodes (RTn) – and chemotherapy. (HRs adjusted for age, comorbidity, cancer stage, grade and ER status).

## Data Availability

The data used in this study are available from the DBCG database and the national medical registries. However, data are only available for the authors due to the legislation of data protection.

## Abbreviations

ATC: Anatomical Therapeutic Classification
CCI: Charlson comorbidity index
CI: Confidence interval
CT: Chemotherapy
DBCG: Danish Breast Cancer Group
DNPreR: Danish National Prescription Registry
DNRP: Danish National Registry of Patients
ER: Oestrogen receptor
RT: Radiation therapy
RTc: Radiation therapy targeting the chest wall/breast only
RTn: Radiation therapy targeting the lymph nodes
HR: Hazard ratio
ICD: International Classification of Diseases
