# Supplementary material for: Incidence of hypothyroidism after treatment for breast cancer—a Danish matched cohort study
Source: Breast Cancer Res. 2020 Oct 13;22:106. doi: 10.1186/s13058-020-01337-z (PMC7556927; doi:10.1186/s13058-020-01337-z)
Supplement: Supplementary file 1 — Additional file 1: Supplementary Figure 1. CONSORT diagram showing the of inclusion and exclusion criteria for the cohort of breast cancer survivors and the matched control cohort. Supplementary Table 1. Incidence rates (IRs) and hazard rates§ of hypothyroidism for survivors of non-metastatic breast cancer and matched controls in strata by calendar period, age and comorbidity at index date. Supplementary Figure 2. Cumulative person-time in the cohort of survivors of non-metastatic breast cancer and the matched control cohort, with that in the matched controls divided by 5 to account for the 1:5 matching. Supplementary Table 2. Incidence rates (IRs) and hazard ratios and associated 95% confidence intervals (95%CI) of hypothyroidism in the cohort of women diagnosed with non-metastatic breast cancer between 1996 and 2009, who were registered in the Danish Breast Cancer Group clinical database, stratified by the receipt of radiation therapy – to the chest wall only (RTc) or with the addition of the lymph nodes (RTn) – and chemotherapy. (HRs adjusted for age, comorbidity, cancer stage, grade and ER status). [file 13058_2020_1337_MOESM1_ESM.docx]

***Supplementary Figure 1. CONSORT diagram showing the of inclusion and exclusion criteria for the cohort of breast cancer survivors and the matched control cohort***

***
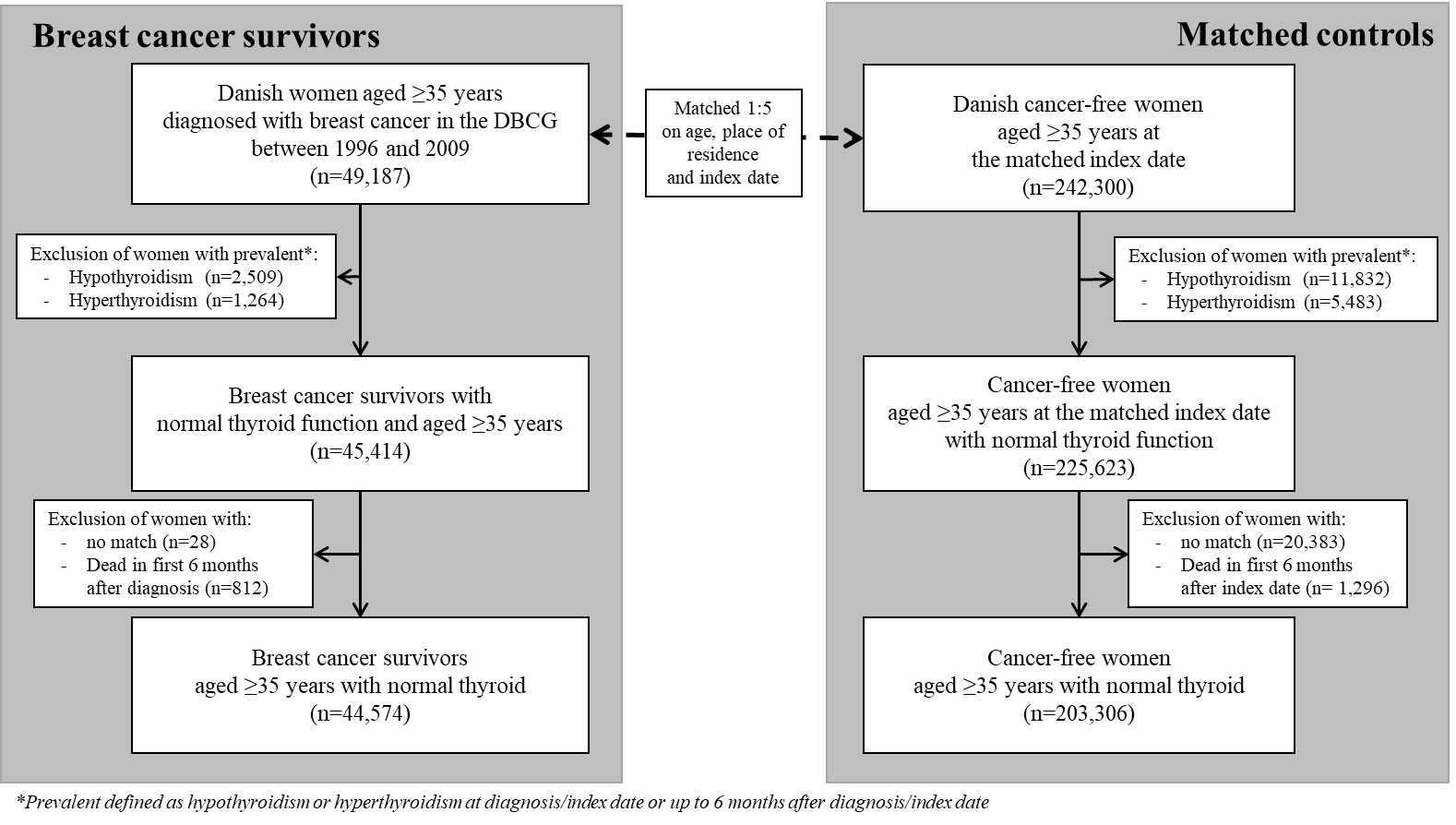
***

| **SUPPLEMENTARY TABLE 1\| Incidence rates (IRs) and hazard rates^§^ of hypothyroidism for survivors of non-metastatic breast cancer and matched controls in strata by calendar period, age and comorbidity at index date.** | | | | | | | | | | | | |
| --- | --- | --- | --- | --- | --- | --- | --- | --- | --- | --- | --- | --- |
|  | | **Breast cancer survivors** | | | | **Matched controls** | | | | **Hazard Ratio** | |  |
|  |  | Cases of hypothyroidism | Numbers | Person years | IR pr. 1000 PY (95% CI*) | Cases of hypothyroidism | Numbers | Person years | IR pr. 1000 PY (95% CI) | Crude (95% CI) | Adjusted (95% CI) |  |
| **Calendar period at index date** | | | |  |  |  |  |  |  |  |  |  |
|  | 1996-1999 | 500 | 11,261 | 122,488 | 4.08 (3.73-4.46) | 2696 | 52,931 | 728,356 | 3.70 (3.56-3.84) | 1.17 (1.06-1.30) | 1.16 (1.05-1.29) |  |
|  | 2000-2003 | 521 | 12,285 | 119,275 | 4.37 (4.00-4.76) | 2457 | 56,836 | 647,295 | 3.80 (3.65-3.95) | 1.12 (1.01-1.23) | 1.11 (1.01-1.22) |  |
|  | 2004-2006 | 354 | 9,458 | 73,570 | 4.81 (4.32-5.34) | 1547 | 42,819 | 380,265 | 4.07 (3.87-4.28) | 1.20 (1.07-1.36) | 1.20 (1.06-1.35) |  |
|  | 2007-2009 | 337 | 11,570 | 69,068 | 4.88 (4.37-5.43) | 1236 | 50,720 | 326,098 | 3.79 (3.58-4.01) | 1.27 (1.13-1.44) | 1.25 (1.11-1.42) |  |
| **Age at index date** | | |  |  |  |  |  |  |  |  |  |  |
|  | 35 to 39 years | 51 | 1,367 | 13,267 | 3.84 (2.86-5.05) | 223 | 6,524 | 77,683 | 2.87 (2.51-3.27) | 1.38 (1.01-1.88) | 1.37 (1.00-1.88) |  |
|  | 40 to 49 years | 317 | 7,123 | 72,100 | 4.40 (3.93-4.91) | 1450 | 34,243 | 402,861 | 3.60 (3.42-3.79) | 1.23 (1.09-1.39) | 1.23 (1.08-1.39) |  |
|  | 50 to 59 years | 529 | 12,543 | 119,198 | 4.44 (4.07-4.83) | 2421 | 59,038 | 659,890 | 3.67 (3.52-3.82) | 1.22 (1.10-1.34) | 1.20 (1.09-1.32) |  |
|  | 60 to 69 years | 486 | 12,945 | 108,906 | 4.46 (4.07-4.88) | 2293 | 58,333 | 575,941 | 3.98 (3.82-4.15) | 1.14 (1.03-1.26) | 1.13 (1.02-1.26) |  |
|  | 70 to 79 years | 255 | 7,704 | 55,466 | 4.60 (4.05-5.20) | 1215 | 33,102 | 289,098 | 4.20 (3.97-4.45) | 1.13 (0.98-1.31) | 1.10 (0.95-1.28) |  |
|  | ≥ 80 years | 74 | 2,892 | 15,464 | 4.79 (3.76-6.01) | 334 | 12,066 | 76,543 | 4.36 (3.91-4.86) | 1.07 (0.81-1.41) | 1.06 (0.80-1.39) |  |
| **Comorbidity** | | |  |  |  |  |  |  |  |  |  |  |
|  | No | 1395 | 35,832 | 325,156 | 4.29 (4.07-4.52) | 6512 | 164,373 | 1,724,421 | 3.78 (3.69-3.87) | 1.15 (1.08-1.22) | 1.15 (1.08-1.22) |  |
|  | Low | 275 | 7,083 | 50,434 | 5.45 (4.83-6.14) | 1185 | 31,600 | 293,807 | 4.03 (3.81-4.27) | 1.36 (1.19-1.56) | 1.36 (1.19-1.56) |  |
|  | High | 42 | 1,659 | 8,810 | 4.77 (3.44-6.44) | 239 | 7,333 | 63,786 | 3.75 (3.29-4.25) | 1.44 (1.01-2.07) | 1.44 (1.01-2.07) |  |
| *CI: confidence interval  ^§^HRs adjusted for Charlson comorbidity index. | | | | | | | | | | | | |

***Supplementary Figure 2. Cumulative person-time in the cohort of survivors of non-metastatic breast cancer and the matched control cohort, with that in the matched controls divided by 5 to account for the 1:5 matching.***


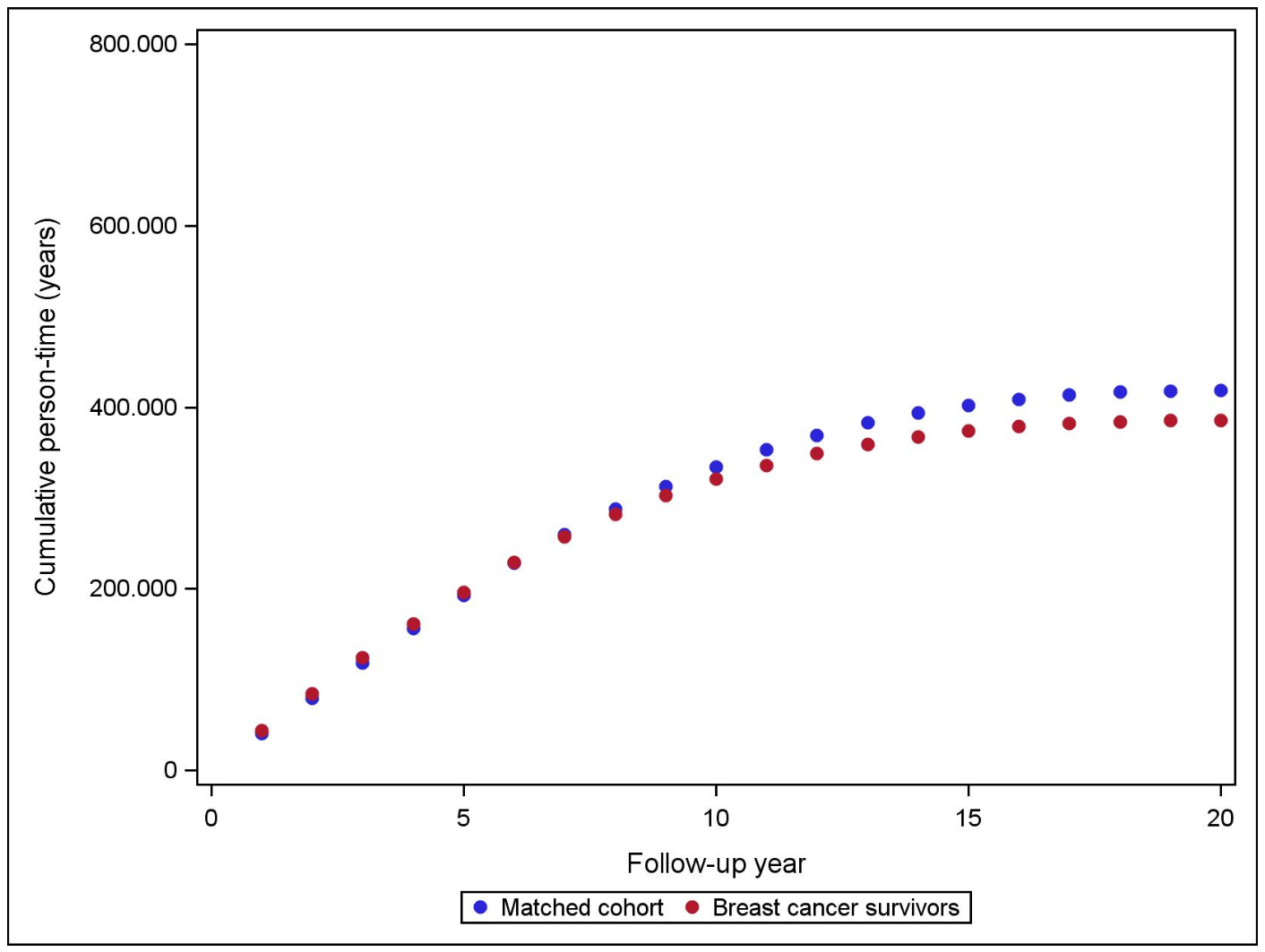


|  | | **SUPPLEMENTARY TABLE 2 : Incidence rates (IRs) and hazard ratios and associated 95% confidence intervals (95%CI) of hypothyroidism in the cohort of women diagnosed with non-metastatic breast cancer between 1996 and 2009, who were registered in the Danish Breast Cancer Group clinical database, stratified by the receipt of radiation therapy – to the chest wall only (RTc) or with the addition of the lymph nodes (RTn) – and chemotherapy. (HRs adjusted for age, comorbidity, cancer stage, grade and ER status).** | | | | | | |
| --- | --- | --- | --- | --- | --- | --- | --- | --- |
|  |  | | **Breast cancer survivors** | | | | **Hazard Ratio** | |
| **TREATMENT MODALITIES** | Median age (years) | | Cases of hypothyroidism | Numbers | Person years | IR pr. 1000 PY  (95% CI) | Crude (95% CI) | Adjusted^1^ (95% CI) |
| RT-/CT- | 66.8 | | 728 | 20,754 | 181,597 | 4.01 (3.72-4.31) | Reference group | |
| RT-/CT+ | 50.1 | | 160 | 4,042 | 40,329 | 3.97 (3.38-4.63) | 0.99 (0.83 - 1.17) | 0.96 (0.77 - 1.21) |
| RTc/CT- | 62.1 | | 245 | 6,615 | 55,188 | 4.44 (3.90-5.03) | 1.12 (0.96 - 1.29) | 1.22 (1.02 - 1.46) |
| RTc/CT+ | 51.8 | | 82 | 2,252 | 19,127 | 4.29 (3.41-5.32) | 1.08 (0.86 - 1.36) | 1.16 (0.88 - 1.53) |
| RTn/CT- | 62.5 | | 254 | 6,065 | 48,237 | 5.27 (4.64-5.95) | 1.32 (1.14 - 1.53) | 1.23 (1.03 - 1.46) |
| RTn/CT+ | 50.1 | | 243 | 4,846 | 39,922 | 6.09 (5.35-6.90) | 1.53 (1.32 - 1.77) | 1.49 (1.22 - 1.82) |
|  | | PY: person years, CI: confidence interval, RT: radiotherapy, CT: chemotherapy, RTc: radiotherapy to the chest wall only, RTn: radiotherapy to the chest wall with addition of lymph nodes (supraclavicular, axillary)  ^1^Adjusted for age, comorbidity at diagnosis, cancer stage, grade, and ER status. | | | | | | |
